# The Effect of Exercise Intervention Based Upon the Selective Functional Movement Assessment in an Athlete With Non-specific Low Back Pain: A Case Report and Pilot Study

**DOI:** 10.3389/fpsyg.2020.02010

**Published:** 2020-08-21

**Authors:** Li Huang, Haowei Liu, Li Zhao, Li Peng

**Affiliations:** ^1^College of Physical Education, Southwest University, Chongqing, China; ^2^Key Lab of Physical Fitness Evaluation and Motor Function Monitoring, Southwest University, Chongqing, China

**Keywords:** Selective Functional Movement Assessment, musculoskeletal disorders, chronic non-specific low back pain, exercise intervention, mobility, stability

## Abstract

**Objectives:**

To illustrate the effectiveness of the Selective Functional Movement Assessment (SFMA) as a guide to exercise intervention on chronic non-specific low back pain (CNLBP).

**Methods:**

A 23-year-old male volleyball athlete with CNLBP was evaluated using the SFMA to assess the degree of physical dysfunctions. And then two-stage exercise protocol was designed based on the results of SFMA. The athlete conducted the exercise intervention for 8 weeks, 1 h each time, three times a week. Transverse abdominal muscles and multifidus muscle thickness, the degree of low back pain, and the degree of physical dysfunctions were measured at pre-intervention, midintervention, and post-intervention.

**Results:**

Based on the results of SFMA, the exercise protocol in the first 4-week session was designed mainly to develop the mobility of ankle, hip, and chest and the stability of lumbar, hip, and knee, in order to improve core strength and gluteal muscle strength. The second 4-week session was an advanced stage with the increase of exercise load on the basis of flexibility and stability; its main purpose was to loosen the hamstring muscles and continue strengthening the core stability and finally help the participant to establish the correct movement pattern and solve the problems of dysfunctions. After 8-week exercise intervention, all movement patterns became functional/non-painful except the deep squat pattern; the Quebec Back Pain Disability Scale score decreased from 11 to 2; visual analog scale score decreased from 4 to 2; the thicknesses of the transverse abdominis muscles (right side: 0.2 vs. 0.31 cm, left side: 0.22 vs. 0.33 cm) and multifidus muscles (right side: 2.09 vs. 2.26 cm, left side: 2.15 vs. 2.29 cm) were both increased.

**Conclusion:**

In this case, the SFMA helped to recognize problems related to mobility and stability on the hip joint, thoracic spine, and even areas far away from the lumbar spine in an athlete with CNLBP that were not seen with more conventional examination procedures. The improvements of physical function, the increase in deep core muscles thickness, and the released pain after exercise intervention all verified the effectiveness of SFMA to qualitatively analyze movement patterns at examination and to direct subsequent exercise intervention.

## Introduction

In many industrialized countries, musculoskeletal disorders (MSDSs) are very common in clinical practice, among which low back pain (LBP) is the most common one. LBP refers to the pain or discomfort in lumbosacral region, with or without lower extremity radiation pain, and is a common health problem in adults, with an incidence of 84% and an increasing trend (Maher et al., 2017). The incidence of LBP increased by 18% between 2006 and 2016 (Global Burden of Disease [GDB], 2016 Disease and Injury incidents and prevalence Collaborators, 2017). LBP not only causes physical dysfunction, but also leads serious health and socioeconomic problems (Foster et al., 2018). In 85–95% of LBP patients, no pathological cause was found, so they were described as having non-specific LBP (NSLBP). Chronic non-specific LBP (CNLBP) is a chronic pain syndrome associated with significant pain in the waist, lumbosacral region, and buttocks and without a clear clinical etiology (Kang et al., 2016), which may cause dysfunction and disability, and affects the quality of human life.

Chronic non-specific LBP patients lack a clear diagnosis or clearly identified anatomic source for their pain, but it can be identified by the presence of aberrant movements, because CNLBP may be a problem of muscle strength, endurance, or neuromuscular control and diminished trunk or lower quadrant strength and endurance. The Selective Functional Movement Assessment (SFMA) in this study is a diagnosis system based on the action mode designed by Cook (2010), mainly used on the evaluation of pain and injury. Compared with other MSDS evaluation tools, SFMA mainly diagnoses and evaluates the motor dysfunction of the subjects and further analyzes and explores the location and causes of the injury (Goshtigian and Swanson, 2016). This evaluation method regards the body as a whole, and each joint is interrelated rather than independent, on the basis of performing its own duties. The pain of a certain joint not only shows that the joint has been damaged to a certain extent, but also may indicate that the function of its related joints has not been properly performed, which then results in the compensatory work of adjacent joints and the occurrence of injury and pain. Although the SFMA is useful with any patient, those with NSLBP are particularly good candidates for evaluation because they lack a clear diagnosis or clearly identified anatomic source for their pain. And one of the mechanisms of NSLBP is movement control impairment, which is defined as the change of spinal alignment and movement pattern in a specific direction, due to the change of joint motion accuracy caused by repetitive movements and long-term postures related to daily activities (Harris-Hayes and Van Dillen, 2009). O’Sullivan (2005) proposed that this is the clinical characteristics of NSLBP patients. She also reported in 2004 that all diseases involving lumbar pain are related to motor or control disorders (O’Sullivan, 2004). Clinicians believe that motor dysfunction syndrome is related to neuromuscular control impairment, which can be identified by clinical observation of abnormal motor patterns (Van Dillen et al., 2003). Biely et al. (2014) experienced physiotherapists in 14 years, observed the trunk movement of 102 subjects, and found that abnormal movement patterns were significantly related to back pain, which provided effective support for the association between abnormal movement patterns and current symptoms. A case report on an 18-year-old athlete with NSLBP evaluated by SFMA found limited mobility of the thoracic and hip joints in the pain-free areas (Goshtigian and Swanson, 2016). It is found that the squat movement pattern in SFMA evaluation results is of great significance to the overall flexibility and stability of the athletes; the squat motion pattern can be used to assess the flexibility and stability of the athlete where motor control dysfunction occurs (Myer et al., 2014). According to the SFMA evaluation of female rugby players (Mckeown et al., 2014), the dysfunction of the athletes in the thoracic and ankle joints was 15% higher than that of other joints. After 1 month of corrective training, the injury rate of the players was reduced from 45 to 30%. Being a clinical assessment system used as a movement-based diagnostic tool to identify musculoskeletal dysfunction by evaluation of fundamental movements, SFMA basically rooted in the theory of regional interdependence (RI), which views all regions of the body as being “musculoskeletally linked” (Sueki et al., 2013). The RI asserts that a patient’s primary musculoskeletal symptom(s) may be directly or indirectly related or influenced by impairments from various body regions and systems regardless of proximity to the primary symptom(s) (Glaws et al., 2014). Seemingly unrelated impairments in remote regions may be the cause of a patient’s reports of pain but may go unidentified if the examination is focused on isolated localized movements alone. RI provides rehabilitation doctors with a methodology to treat pain and dysfunction (Wainner et al., 2007), with treatment not only on the pain area any more. It is a good way to manage musculoskeletal pain, and it can find the root cause of chronic musculoskeletal pain accurately and effectively (Goshtigian and Swanson, 2016).

Exercise therapy is the preferred way for the management of CNLBP to improve dysfunction and alleviate pain in patients (Airaksinen et al., 2006). But the effects of many exercise therapy are not identified, and typically focusing on a single pathological structure often results in poor outcomes (Van Tulder et al., 1997). The new idea of exercise therapy should be to create a new program, which will solve the problem of patients’ dysfunction as a part of each individual customized rehabilitation program. It is important to deal with potential muscle imbalances and lack of movement, which can help patients improve their physical function and reduce the risk of injury. Special requirements for exercise therapy on CNLBP are needed in the methods, contents, and strategies. Studies have successfully linked limitations in remote regions to symptoms elsewhere in the system, including limitations of hip mobility to LBP and foot dysfunction causing patellofemoral pain. These correlations suggest the need for a valid evaluative system capable of identifying these dysfunctions to improve outcomes and potentially decrease recurrence. However, most studies still only consider the impact of hip area on LBP (Murray et al., 2009; Reiman et al., 2009; Burns et al., 2018) and not explicitly suggest that treating the problem of the distal area can improve dysfunction and release pain in patients with CNLBP (Bernet et al., 2019). At the same time, only a very few literature reported the application of SFMA (Goshtigian and Swanson, 2016), but no detailed evaluation on an individual basis and no individualized corrective training program based on the individual after evaluation. Then the purpose of this case report is to describe the evaluation findings of SFMA, design exercise program, and observe the results of intervention in a CNLBP athlete and to highlight the value of SFMA on examination, evaluation, and intervention training for CNLBP.

## Materials and Methods

### Case Description

The participant was a 23-year-old male student at a sports college, majoring in volleyball. He self-reported that the LBP on both sides of L_3_–L_4_ had lasted for about 5 years since his senior high school. At present, there was only slight pain when keeping sitting and bending over in daily life. The LBP was become more obvious after the training classes, especially after jumping and spiking. He had not seen a physician for his LBP or had other consultations or interventions. The participant signed informed consent and corrective training instructions to allow the use of his personal information for this case report and promise to follow the direction of exercise intervention. The case report was approved by the ethics committee of Southwest University (approval no. SWU-20180201-C1).

### Methods

The screening of the subject with CNLBP was performed 1 week before exercise intervention. Medical examination, the visual analog scale (VAS), Quebec Back Pain Disability Scale (QBPDS), and SFMA were taken 2 days before intervention, and 2 days after the mid-session and last intervention session. The participant performed 8-week exercise intervention based on the results of SFMA for 1 h each time and three times a week. During the intervention training, the patient was not asked to change his way of life and behavior, and he maintained his usual state of life including daily activity and a volleyball class. The Volleyball class is a professional course, twice a week and two sessions ×40 min each time with a 10-min interval between sessions.

### Medical Examination

Besides physical examination, the x-ray examination and musculoskeletal ultrasound of lumbar spine alignment, vertebral body and intervertebral disk space, bone space and structure, and soft tissue structure were made.

Transverse abdominal muscle and multifidus muscle thickness was assessed using a 5- to 18-Hz probe (Samsung HM70A). The accuracy is 0.01 cm. Transverse abdominal muscles were acquired while patient was resting in a supine position on a table, and these images were acquired from the left and right side, with the probe positioned along the midaxillary line, approximately halfway between the iliac crest and the inferior border of the rib cage. Lumbar multifidus muscle were acquired while patient was resting in a prone position on a table, and the lumbar multifidus muscle at the L_4_–L_5_ levels were measured as the distances between the most superficial portion of the facet joints and the plane between the muscle and subcutaneous tissue (Nuzzo and Mayer, 2013).

### Selective Functional Movement Assessment

The subject was assessed by testers with SFMA professional certification via the SFMA top-tier patterns in order to identify functional movement deficits. It is evaluated from the aspects of motion mode, body function and pain, and mainly including seven movement patterns: (1) cervical patterns (flexion, extension, right and left rotation), (2) upper extremity [medical extension–rotation patterns (MRE)], (3) upper extremity [lateral rotation abduction pattern (LRA)], (4) multisegmental patterns, (5) multisegmental rotation patterns, (6) single leg stance, (7) deep squat.

In order to facilitate the record, the combination of four letters F (function), D (dysfunction), P (pain), and N (non-pain) is generally used for combined assessment results as FN, FP, DN, and DP. FN means that the movement can be completed at standard and without pain; FP means that the movement can be completed at standard but with a certain degree of pain; DN means that the movement cannot be completed at standard but no pain; DP means that the movement cannot be completed and with pain (Cook, 2010).

### Quebec Back Pain Disability Scale

The QBPDS includes 20 self-reported items to assess the level of functional disability in individuals with LBP, designed by Kopec et al. (1995). All the items are grouped into six categories: bed/rest (items 1–3), sit/stand (items 4–6), walking (items 7–9), movement (items 10–12), bending (items 13–16), and handle heavy objects (items 17–20). There six options were scored from 0 to 5 points for each item, indicating the level of difficulty in completing the activity; the higher the score, the more severe the dysfunction.

### Visual Analog Scale

The VAS was used to describe the patient’s objective pain level, as is suitable as a common tool for self-assessment of pain. The VAS is a linear scale from 0 to 10 cm, representing different pain levels; the higher the score, the more severe the pain.

The information on comprehensive subjective and physical examination is summarized in Table 1.

**TABLE 1:** Subjective and physical examination findings.

23-year-old male; unmarried
Work: college student, majoring in volleyball
Home: household activities; sitting and doing work with computer
History: starting during the his high school
Pain: LBP only (both sides of L_3_–L_4_)
Aggravating postures: sitting on a hard chair; sustained forward bending; sustained backward bending
Aggravating activities: doing heavy squat; jumping; running
Pain intensity (VAS): 4/10 (ordinary life activities)
Disability score (QBPDS): 11
Medical imaging (x-ray): no abnormalities detected
Serious pathology (“red” flags): absent

### Exercise Protocol

The intervention was composed of two exercise protocol sessions. To begin every exercise session, the subject was assessed using each of the SFMA top tier movements in order to guide exercise protocol (Kolar et al., 2012). Limited motion or soft tissue extensibility was addressed using sustained stretching including rear foot elevated hip flexor stretches and contract relax stretching on the hamstrings, respectively, for 30 s each time. According to the initial result of SFMA, the program in the first 4-week session was designed mainly to develop the mobility of ankle, hip, and chest and the stability of lumbar, hip, and knee, in order to improve core strength and gluteal muscle strength (Wu, 2018). In the second 4-week session, the exercise program was adjusted according to the results of SFMA in the midterm. The main purpose was to loosen the hamstring muscles and continue strengthening the core stability and finally help the patient to establish the correct movement pattern. In addition, according to the DN result of deep squat test, establish squat movement, lunge movement, and deadlift movement patterns were added to the exercise protocol. The second session was an advanced stage, with the increase of exercise load on the basis of flexibility and stability exercises. And the movements chosen into exercise protocols were mainly from the official website of Functional Movement^1^. Each movement was conducted as 12–15 repetitions × three sets each time, with 30- to 60-s intervals between sets.

## Results

### SFMA Results

The initial results of the SFMA top-tier screening showed 5 DN and 1 DP in seven movement patterns, a detailed intuitive image of which can be found in Appendix A. After the first stage of training, only multisegmental flexion, right-leg stance, and deep squat were still DN; multisegmental rotation has changed from DN on the left and right sides to FN, and the DP problem of multisite stretching has been solved. After the second stage of intervention, multisegmental rotation flexion and one-leg stance on the right leg have been improved to FN, and only deep squat remained DN (Table 2).

**TABLE 2:** Results of SFMA at pre-intervention, mid-intervention, and post-intervention.

Assessment of movement patterns			0 week	4 weeks	8 weeks
Cervical		Flexion	FN	FN	FN
		Extension	FN	FN	FN
Cervical rotation		Left	FN	FN	FN
		Right	FN	FN	FN
Upper extremity	MRE	Left	FN	FN	FN
		Right	FN	FN	FN
	LRA	Left	FN	FN	FN
		Right	FN	FN	FN
Multisegmental		Flexion	**DN**	**DN**	FN
		Extension	**DP**	FN	FN
Multisegmental rotation		Left	**DN**	FN	FN
		Right	**DN**	FN	FN
Single leg stance		Left	FN	FN	FN
		Right	**DN**	**DN**	FN
Deep squat			**DN**	**DN**	**DN**

*DN = Dysfunctional/Non-Painful, DP = Dysfunctional/Painful, FN = functional/Non-painful. The bold font can be easily identified the dysfunctional movement patterns.*

### Scores of QBPDS

**FIGURE 1:**
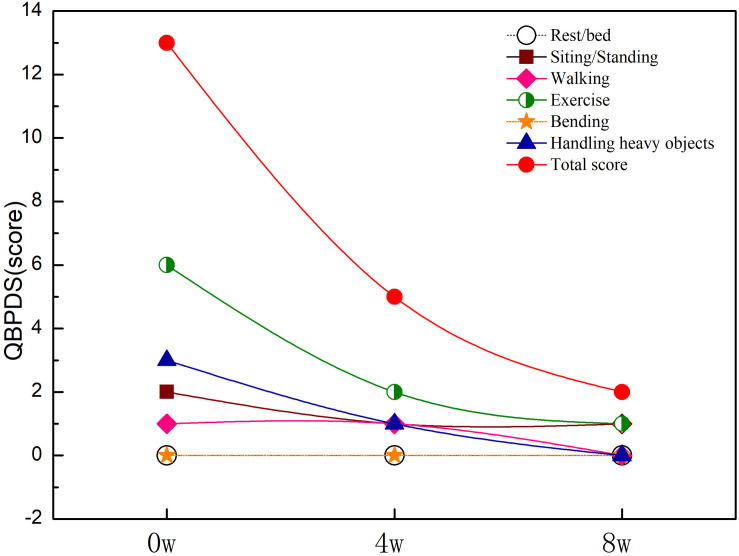
QBPDS score at pre-intervention, midintervention, and post-intervention.

As can be seen in Figure 1, the initial results of the QBPDS revealed 0 point for rest/bed, 2 points for sitting/standing, 2 points for walking, 6 points for exercise, 0 point for bending, and 3 points for handling heavy objects, for 13 points in total. The dysfunction in daily life is shown, especially when handling heavy objects and doing exercise. After 8 weeks of exercise intervention, the total QBPDS score of the participant decreased from 11 to 2 points. After the first stage of exercise, the dysfunction in terms of exercise and handling heavy objects improved significantly; the score for exercise capacity was reduced from 6 to 2; the score to handle heavy objects was reduced from 3 to 1, and the score for sit/stand was reduced from 2 to 1, walking score was still 1 point. After the second stage of exercise, there was no dysfunction except 1 point for both sitting/standing and exercise.

### Scores of VAS

**FIGURE 2:**
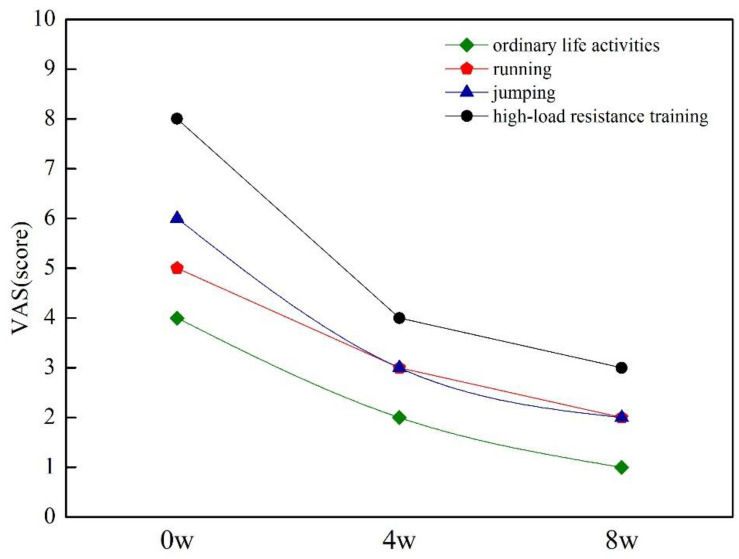
VAS score at pre-intervention, midintervention, and post-intervention.

Before the intervention, the patient reported 4/10 in ordinary life activities; 5/10 when running more than 400 m; 6/10 during volleyball training, especially when jumping; and 8/10 when performing high-intensity resistance training. After 8 weeks of training, the subject total LBP VAS score decreased from 4 to 2 (Figure 2). Through the first stage of exercise, the pain level reduced as 2/10 in ordinary life activities, 3/10 when running or jumping, and 5/10 when performing resistance training. According to the patient’s description, the LBP released obviously after weight-bearing fitness and volleyball training classes and when sitting or standing for a long time in daily life. After the second stage of exercise, there was no obvious pain after the volleyball class (2/10); the pain also disappeared when sitting or standing for a long time in daily life (1/10), and only a long period of weight-bearing training can cause a slight pain (4/10) (Figure 2).

### Results of Medical Examination

Before the intervention, the x-ray examination showed there was no un-normal part in lumbar spine alignment, vertebral body and intervertebral disk space, bone space and structure, and soft tissue structure. The gait was stable, coordinated, and rhythmic, and the legs were alternated; there was physiological curvature of the lower back; there were no side bends, hump, no pain in the hips and thighs, and normal lower limb sensation and reflection, as the results of physical examination.

**FIGURE 3:**
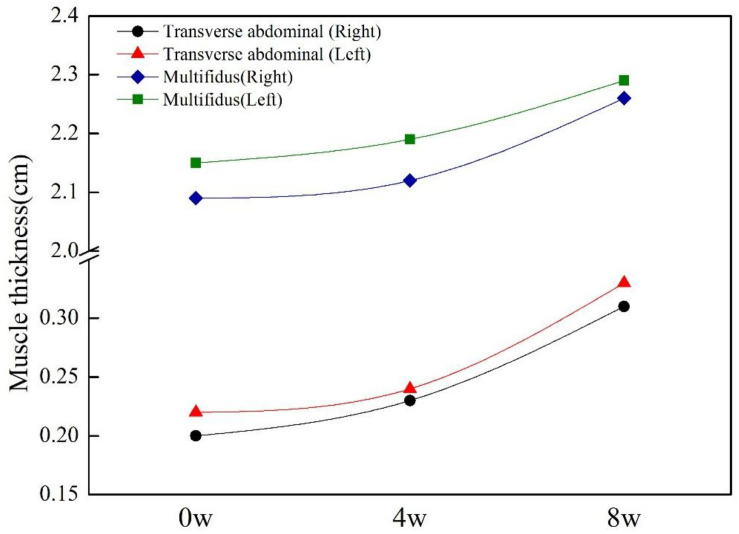
Muscle thickness at pre-intervention, midintervention, and post-intervention.

The initial results showed the thicknesses of the transverse abdominal muscles of the participant were 0.20 cm (right) and 0.22 cm (left), and the thicknesses of the L_4_–L_5_ multifidus muscle were 2.09 cm (right) and 2.15 cm (left), a detailed intuitive image of which can be found in Appendix B. After 8 weeks of corrective training, the thicknesses of the transverse abdominis muscles increased to 0.31 cm (right) and 0.33 cm (left), and the thicknesses of multifidus muscles increased to 2.26 cm (right) and 2.29 cm (left) (Figure 3).

## Discussion

Pain is a subjective experience that results from the modulation of nociception conveyed to the brain via the nervous system. Perception of pain takes place when potential or actual noxious stimuli are appraised as threats of injury. This appraisal is influenced by one’s cognitions and emotions based on his/her pain-related experiences, which are processed in the forebrain and limbic areas of the brain. Unarguably, patients’ psychological factors such as cognitions (e.g., pain catastrophizing), emotions (e.g., depression), and pain-related behaviors (e.g., avoidance) can influence perceived pain intensity, disability, and treatment outcomes (Hamasaki et al., 2018). Fear avoidance behavior related to physical activity and work is one of the causes of CLNBP. In this case study, the possibility of fear avoidance behavior cannot be ruled out completely, but it is highly unlikely, because the patient in this case study insists on volleyball training for many years under the condition of LBP. The degree of pain in different living conditions was measured by VAS and found that his LBP was closely related to sports training. Therefore, as an athlete’s LBP, it is considered that dysfunction has a greater impact. And the results of SFMA verified the original judgment: the patient may suffer from LBP due to dysfunction of other parts of the body or may suffer from long-term LBP, which affects the function of other parts of the body. The initial results of SFMA were five DN and one DP. Dysfunctional movement in these patterns can suggest mobility limitations, stability dysfunction, or both. Mobility limitations were affected by tissue extensibility or joint mobility dysfunction. Stability dysfunctions also referred to “motor control dysfunction,” which is complex and affected by multiple factors or systems, not only the strength of the stabilizer muscles but also the central and peripheral nervous systems, the proprioceptive system, postural alignment, structural integrity, and muscular inhibition (Goshtigian and Swanson, 2016). Mobility and stability limitations frequently coincide, as the body may sacrifice mobility in one region in an attempt to achieve a compensatory “pseudostabilization” in another (Cook, 2010). According to the initial results of SFMA, it was found that the main problems of the participants were tension of hamstring, quadriceps, iliopsoas, and iliotibial tract, limitation of ankle dorsiflexion function, and lack of core stability. Therefore, aiming at the lack of flexibility of hip and ankle and weakness of core stability, the exercise intervention protocol was designed. And the final results of SFMA showed only deep squat remaining DN; the QBPDS and VAS scores decreased obviously; almost all the dysfunction problems have been solved, which proved the success of the exercise scheme based on SFMA results. The problems of lack of flexibility of hip and ankle joints were solved, and the strength of core muscles and reconstruction of the correct movement patterns were enhanced through exercise intervention in this study, which helped to stabilize and enhance the core stability, so as to alleviate the LBP. It is essential for musculoskeletal problems to improve the flexibility and stability and maintain a balance between them. When the mobility of the adjacent joints of the lumbar vertebrae is insufficient, especially when the mobility of the hip joint is insufficient, the lumbar vertebrae will compensate, thereby decreasing its stability function and causing LBP (Shuyan et al., 2018). There are also studies in enhancing the core stability by improving hip function and hip muscle strength. The strength of abductor muscle group of hip joint was strengthened in three elderly patients with CNLBP, which improved the waist dysfunction and released pain (Peterson and Denninger, 2017). Low back pain was relieved by performing hip traction and strength training in 30 patients with CNLBP (Winter, 2015).

In the core muscles of trunk, respiratory muscle, abdominal oblique muscle, abdominal transverse muscle, and multifidus muscle are the important muscles that affect the stability of spine. Of all the abdominal muscles, the transverse abdominal muscle and thoracolumbar fascia are the widest and most closely connected and play a role in stabilizing the lumbar spine and controlling the abdominal pressure. Therefore, it is essential for the stability of the spine (Marshall et al., 2012). Compared with healthy subjects, the abdominal muscle thickness of patients with LBP decreased, and it is pointed out that the pain level and disability of patients with LBP were significantly negatively correlated with the abdominal muscle thickness (Rahmani et al., 2018). Biomechanics studies suggest that the role of multifidus muscle is to provide lumbar vertebral stiffness (Kim and Kwon, 2012), control the neutral area of the spinal section, and maintain lumbar vertebrae stability when challenged on stabilization (Debuse et al., 2013). LM atrophy in LBP patients has been confirmed by several studies (Hides et al., 1994), and the thickness of deep muscles of the torso is often asymmetric (Stokes et al., 2005). The recruitment of abdominal transverse muscle and multifidus muscle decreased significantly in patients with LBP, and the thicknesses of abdominal transverse muscle and multifidus muscle in NLBP patients were significantly lower than those in healthy people, indicating that the patients with NLBP may be accompanied by atrophy of abdominal transverse muscle and multifidus muscle at the same time. In this study, the thicknesses of the transverse abdominal muscle and multifidus muscle were measured before and after exercise intervention by using musculoskeletal ultrasound. Ultrasound imaging measurements are adequately reliable to assess muscle thickness; even novice raters who complete a standardized training program can reliably obtain these measurements (Wallwork, 2007). The interrater reliability point estimates of 0.80–0.94 were used to assess lumbar multifidus muscle thickness (Koppenhaver et al., 2009). A single-group repeated-measures reliability study was conducted on 21 healthy participants without LBP; ultrasound images of the transversus abdominis and lumbar multifidus muscles were obtained by different pairs of novice raters in a counterbalanced order, and it was found that thickness measures of the transversus abdominis and lumbar multifidus muscles obtained with ultrasound imaging at rest and during submaximal contractions demonstrate good to excellent reliability. These results support the potential use of ultrasound imaging measurements for management decisions in the diagnosis and treatment motor control impairments. The mean minimal important difference of transversus abdominis and the lumbar multifidus muscle thickness was 15 and 42 mm, respectively (Teyhen et al., 2011). The results of this current study showed only an inconspicuous increase in the thicknesses of the transverse abdominal muscle (11 mm) and multifidus muscle (15.5 mm) according to the minimal important difference value of Deydre’s study, but it did reach the result of another study that also looked at Chinese people, 5.5 mm of the mean minimum detectable change value when using the ultrasound imaging technology to measure abdominal muscle thickness (Chunlong et al., 2014). However, the minimal important difference value of transversus abdominis thickness and the lumbar multifidus muscle thickness of the two studies were from healthy persons, and the data from CLNBP have not been accessed. One purpose of exercise rehabilitation on NSLBP is to correct asymmetry through specific exercises to enhance the stability of lumbar vertebral segments. If the intervention can increase the thickness of the multifidus and transverse abdominal muscles, it is a good signal to improve the pain and function of patients with NSLBP. From the perspective of the change rate of muscle thickness, the change rate of the first-stage intervention was generally less than the second stage, as shown in Figure 3. The reason may be that most of the training movements in this study were re-education of neuromuscular system with a few low-load activation training. The first 4-week training was mainly to increase the recruitment of stable muscles. Studies have shown that the acquisition of most early strength is more neural rather than contractile (Boyle, 2014). Therefore, the increase of the thickness of the transverse abdominal muscles and multifidus muscles in this case is conducive to maintaining the stability of the lumbar spine, possibly by enhancing neuromuscular control to reduce pain and dysfunction scores (Ferreira et al., 2006).

## Limitations

Typical of case reports, the single-subject design limits a cause-and-effect relationship. Interventions based on the SFMA can vary greatly, as intervention choices are dependent on practitioner judgment, experience, and personal equipoise. As a result, a definite limitation of application of this exercise protocol and only an effective framework for the evaluation and treatment of a patient with CNLBP was presented.

## Implications

The NSLBP case in our study had hip flexibility disorder or stability/motor control disorder, which was consistent with the results from other studies. The exercise training for NSLBP in the future should focus on the flexibility and motor control of hip and introduce the concept of the lumber–pelvis–hip complex into the training; the functional disorder of other joints such as ankle, knee, and thoracic vertebrae also needs to be considered. In the design of exercise protocols, training on proprioception including the acceleration and deceleration ability and the control ability in unstable state can be adopted. And an extended follow-up study should be conducted to observe the long-term effect.

## Conclusion

In this case, the SFMA helped to recognize problems related to mobility and stability on the hip joint, thoracic spine, and even areas far away from the lumbar spine in an athlete with CNLBP that were not seen with more conventional examination procedures. The results of exercise intervention showed the improvement of physical function, the increase of thickness of deep core muscles, and the released LBP, which verified the effective use of SFMA to analyze movement patterns at examination qualitatively and to direct subsequent exercise intervention.

## Data Availability Statement

All datasets generated for this study are included in the article/supplementary material.

## Ethics Statement

The studies involving human participants were reviewed and approved by the Hospital Ethics Committee of the Southwest University in China. The patients/participants provided their written informed consent to participate in this study. Written informed consent was obtained from the individual(s) for the publication of any potentially identifiable images or data included in this article.

## Author Contributions

LP was responsible for experimental design and financing. HL and LH were in charge of SFMA and questionnaires, data collection, and exercise scheme design. LZ was responsible for medical examination. HL was in charge of guiding the exercise intervention. LH was in charge of thesis writing. All authors contributed to the article and approved the submitted version.

## Conflict of Interest

The authors declare that the research was conducted in the absence of any commercial or financial relationships that could be construed as a potential conflict of interest.

## Footnotes

1 www.functionalmovement.com
